# Selection of a novel CD19 aptamer for targeted delivery of doxorubicin to lymphoma cells

**DOI:** 10.18632/oncotarget.24902

**Published:** 2018-06-01

**Authors:** Yan Hu, Xiaoou Li, Yacong An, Jinhong Duan, Xian-Da Yang

**Affiliations:** ^1^ Institute of Basic Medical Sciences, Chinese Academy of Medical Sciences & Peking Union Medical College, Beijing 100005, China

**Keywords:** CD19, aptamer, targeted therapy, lymphoma

## Abstract

CD19 is overexpressed in most human B cell malignancies and considered an important tumor marker for diagnosis and treatment. Aptamers are oligonucleotides that may potentially serve as tumor-homing ligand for targeted cancer therapy with excellent affinity and specificity. In this study, we selected a novel CD19 aptamer (LC1) that was a 59-nucleotide single strand DNA. The aptamer could bind to recombinant CD19 protein with a *K*_d_ of 85.4 nM, and had minimal cross reactivity to bovine serum albumin (BSA) or ovalbumin (OVA). Moreover, the aptamer was found capable of binding with the CD19-positive lymphoma cells (Ramos and Raji), but not the CD19-negative cell lines (Jurkat and NB4). An aptamer-doxorubicin complex (Apt-Dox) was also formulated, and selectively delivered doxorubicin to CD19-positive lymphoma cells *in vitro*. The results indicate that aptamer LC1 can recognize CD19-positive tumor cells and may potentially function as a CD19-targeting ligand.

## INTRODUCTION

Hematologic malignancies are serious threat to human health and account for an estimated 6.5% of new cancer cases worldwide, with rising incidence in recent years [1]. Among hematologic malignancies, lymphoma is the most prevalent type and responsible for 55.6% of the cases. Conventional treatment of lymphoma usually consists of multi-drug chemotherapy, but the efficacy of chemotherapy is often limited by drug-resistance and adverse effects associated with cytotoxic agents. One strategy to improve the outcome of lymphoma treatment is targeted therapy, which can direct the anti-cancer firepower to tumor cells and minimize the damage to normal tissue. Targeted therapy generally requires a therapeutic target, which is usually a tumor marker that is overexpressed on the surface of cancer cells but underexpressed in normal tissue. Moreover, it is also necessary to have a tumor-targeting ligand, which can recognize the tumor marker and bring antitumor drugs or lymphocytes to the cancer cells, to achieve the targeted therapeutic effects.

CD19 is an important therapeutic target for treatment of lymphoma and lymphocytic leukemia. It is a 95 KD transmembrane glycoprotein that is overexpressed in most B cell lymphomas, B-cell acute lymphocytic leukemia, and B-cell chronic lymphocytic leukemia [2, 3]. Since nearly 80-85% of lymphomas originate from B-cells, and approximately 88% of these cells reliably express surface CD19 [4], targeting CD19 is a promising strategy for treatment of lymphocytic malignancies. Recently, targeted immunotherapy aiming at CD19, with chimeric antigen receptor-modified T cells (CAR-T), generated breakthrough results in treatment of acute lymphocytic leukemia, as well as promising outcomes in treatment of chronic lymphocytic leukemia and Non-Hodgins lymphoma [5]. In a recent clinical trial conducted at Fred Hutchinson Cancer Center, 27 of 29 (93%) ALL patients treated by CAR-T therapy achieved BM remission as determined by flow cytometry. Moreover, 25 of the 29 patients (86%) achieved complete remission without evidence of minimal residual disease [6]. These results demonstrated that CD19 is an important therapeutic target with great clinical potential for treating lymphocytic malignancies. At present, CD19-targeted therapy was mainly achieved by antibody-based technology. Here the antibody (or its single-chain variable fragments) serves as the tumor-targeting ligand for recognizing the CD19 molecules on cell surface, and brings anticancer drugs or T lymphocytes to tumor cells [7, 8].

In addition to antibodies, aptamers may also function as tumor-homing ligand for targeted cancer therapy [9]. Aptamers are short single-stranded DNAs, RNAs, or modified nucleic acids that can form self-tertiary structures and bind to designated molecular targets. Aptamers are usually selected from a large pool of nucleic acids through a process called systematic evolution of ligands by exponential enrichment (SELEX). Aptamers can bind to a great variety of targets such as organic and inorganic molecules, peptides and proteins, viruses and cells [10]. Similar to antibodies, aptamers can bind to targets with high affinity and specificity. Compared to antibodies, aptamers have some unique properties. Aptamers can be easily produced through chemical synthesis. The molecular weight of aptamers are significantly lower than antibodies. Aptamers also show low immunogenicity and toxicity *in vivo*. Moreover, aptamers can be readily modified into various functional formats such as aptamer-drug conjugates, aptamer-nanomaterial conjugates, aptamer-fluorescence probes etc. [11–13]. Previous studies have shown that these functional aptamers are quite effective in cancer diagnosis and targeted therapy. Taken together, the features of aptamers make them ideal alternatives to antibodies for cancer research, diagnosis, and therapy.

To date, several tumor-aiming aptamers have been identified and developed, including aptamers targeting MUC1, CD30, HER2, and nucleolin [14–19]. These aptamers have been employed to build selective drug delivery systems for targeted therapy against tumor cells [20–22]. Nevertheless, it technically challenging to select a functional aptamer that can bind to tumor cells with certain degree of specificity and affinity. The clinical importance of CD19 as a therapeutic target has become evident only in recent year with the breakthrough progress in CAR-T therapy. So far, however, aptamers against CD19 have not been reported in literature. In this study, we developed the first CD19 aptamer using SELEX technology, and evaluated its binding to CD19-positive lymphoma cells and CD19-negative control cells. Specifically, a 59-base DNA aptamer (termed LC1) against the extracellular domain of a recombinant CD19 protein was selected. Our data showed that the aptamer could preferentially bind to CD19 protein and CD19-positive lymphoma cells. To investigate whether the aptamer could carry cytotoxic drug to CD19-positive lymphoma cells, an aptamer-doxorubicin complex (Apt-Dox) was formulated. We now report that Apt-Dox selectively delivered doxorubicin to CD19-positive lymphoma cells *in vitro*. The results indicate that aptamer LC1 can recognize CD19 protein and may potentially serve as a tumor-targeting ligand for lymphoma diagnosis and treatment.

## RESULTS

### Aptamer selection

**Figure 1:**
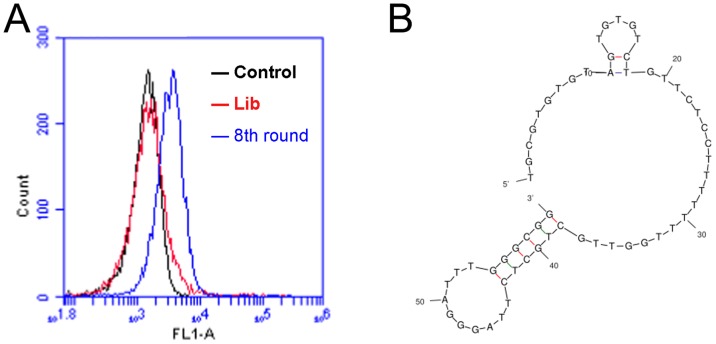
Flow cytometry monitoring of the enrichment of aptamers during selection **(A)** Compared with the initial random DNA pool (the red curve), flow cytometry revealed an increase in fluorescence intensity of DNAs bound to the CD19 protein in the 8th (the blue curve) rounds of selection. **(B)** The predicted secondary structure of aptamer LC1.

The CD19 aptamer was selected using a standard SELEX protocol. Briefly, the extracellular domain of recombinant human CD19 protein was covalently conjugated to magnetic beads. The protein-coated beads were incubated with a random DNA pool and washed. The DNAs absorbed by protein-coated beads were amplified by PCR, and used for next round selection. Flow cytometry was used to monitor the enrichment of target-binding DNAs after each selection round. After eight rounds of selection, increasing fluorescence signals were observed, indicating that more ssDNA bound to the target-coated beads (Figure 1A). This pool of DNA was subsequently cloned. Among the 96 clones analyzed, one aptamer (termed LC1) showed relatively high binding capacity to the target CD19 protein. The primary sequence of aptamer LC1 is TGCGTGTGTAGTGTGTCTGTTCTCCTTTTTTTGGTTGCTGCTCTTAGGGATTTGGGCGG. The predicted secondary structure of LC1 was shown in Figure 1B.

### Binding specificity of aptamer LC1

**Figure 2:**
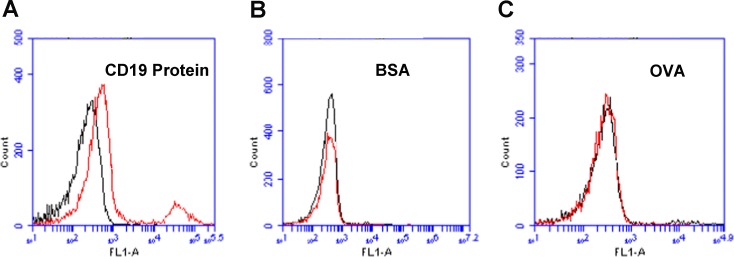
Flow cytometry assessments of the aptamer's binding to CD19 protein, BSA, or OVA **(A)** Beads coated with CD19 protein was treated with FITC-labeled aptamer. **(B)** BSA beads treated with FITC-labeled aptamer. **(C)** OVA beads treated with FITC-labeled aptamer. Black curves represent the control fluorescence signals generated by FITC-labeled random DNA from the library pool. Red curves are signals produced by aptamer LC1.

To evaluate whether aptamer LC1 had a preferred binding with CD19 protein, we compared the aptamer's binding to CD19, BSA, or OVA, under the same experimental conditions. The latter two structures (BSA and OVA) were often used for testing the binding specificity of aptamers [23, 24]. As shown in Figure 2, the aptamer generated a strong binding to CD19 (Figure 2A), but relatively weak bindings to BSA (Figure 2B) or OVA (Figure 2C). The data suggested that aptamer LC1 had a targeting preference towards CD19, and tended not to bind with other structures such as BSA or OVA.

### Aptamer LC1 selectively recognized CD19-positive lymphoma cells

**Figure 3:**
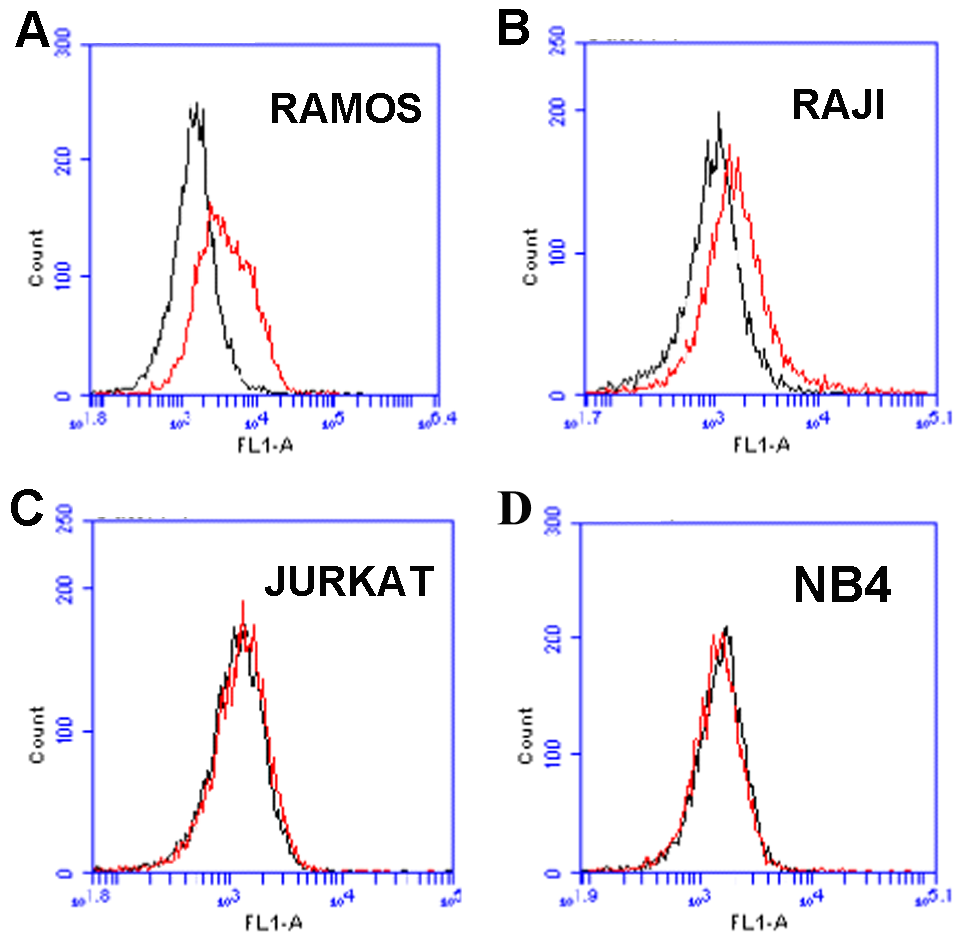
Flow cytometry evaluation of the aptamer's binding to CD19-positive and -negative cells The histograms (red curves) were generated after incubating FITC-labeled aptamer with CD19-positive cells Ramos **(A)**, Raji **(B)**, and the CD19-negative cells Jurkat **(C)**, and NB4 **(D)**, respectively. The black curves represent the control fluorescence signals generated by FITC-labeled random DNA from the library pool.

The above data showed that aptamer LC1 could bind with CD19 protein conjugated to magnetic beads. However, it is still unclear whether the aptamer would recognize the CD19-positive lymphoma cells. To address this issue, CD19-positive lymphoma cells (Ramos and Raji) or CD19-negative cells (Jurkat, and NB-4) were incubated with FITC-labeled aptamer and analyzed by flow cytometry. The expression of CD19 in these cell lines had been extensively analyzed by prior studies with mRNA or western blot assays [25, 26]. As shown in Figure 3, The aptamer LC1 had relative strong bindings to the CD19-positive lymphoma cells Ramos (Figure 3A) and Raji (Figure 3B), whereas its bindings to the CD19-negative cells (Jurkat, and NB-4) were quite weak (Figure 3C & 3D). The data indicated that aptamer LC1 could recognize and preferentially bind with the CD19-positive lymphoma cells *in vitro*.

**Figure 4:**
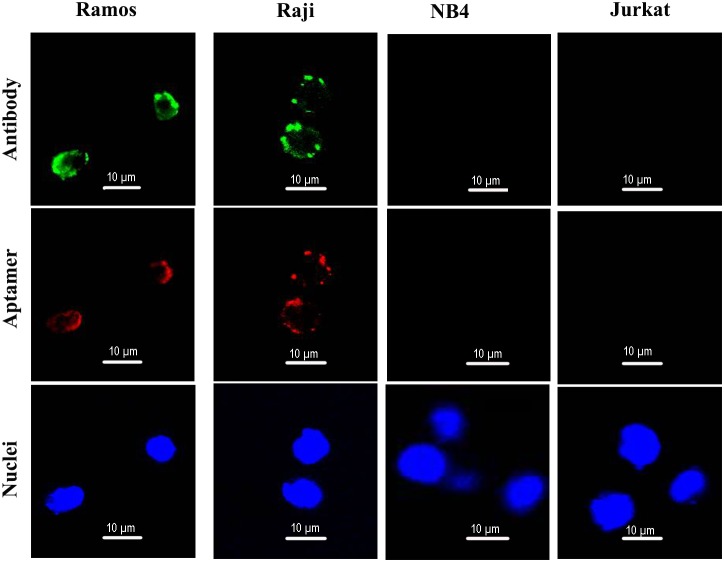
Confocal microscopy evaluation of aptamer's binding to CD19-positive and -negative cells Ramos and Raji are CD19-positive cell lines. NB4 and Jurkat are CD19-negative cells. Green fluorescence signal was generated by Alexa Fluor 488-labeled CD19 antibodies. Red fluorescence signal was generated by Cy3-labeled aptamers. The nuclei were stained blue with DAPI.

To further evaluate whether aptamer LC1 would bind with CD19-expressing lymphoma cells, confocal microscopy was also applied to monitor the fluorescence signals generated by the above four cell lines incubated with Cy3-labeled aptamer. To evaluate CD19 expression by the cell lines, the cells were also stained with anti-CD19 antibody. As presented in Figure 4, Ramos and Raji cells generated strong green fluorescent signals, while Jurkat and NB-4 cells failed to do so, confirming that the lymphoma cells Ramos and Raji cells indeed express CD19. The red fluorescence signals from Cy3-labeled aptamer were remarkably strong in CD19-positive Ramos and Raji cells, but weak in CD19-negative cell lines Jurkat and NB-4, again indicating that the aptamer could selectively bind with CD19-positive lymphoma cells *in vitro*.

### Binding affinity of the aptamer

**Figure 5:**
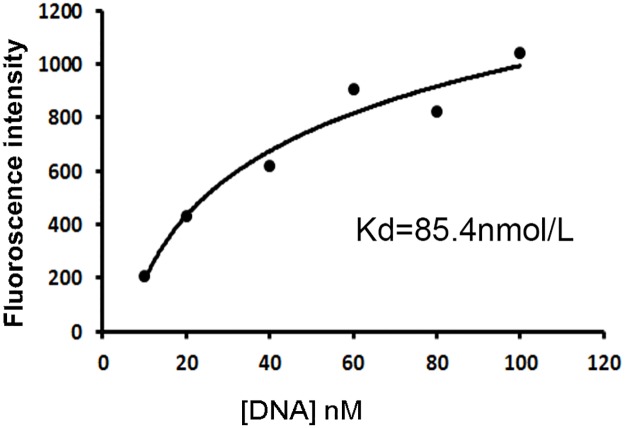
Quantitative evaluation of the aptamer's affinity to CD19 protein FITC-labeled aptamers of various concentrations were incubated with beads coated with the CD19 protein, which were analyzed by flow cytometry. The *K*d was estimated by plotting the reciprocal of fluorescence intensity against the reciprocal of DNA concentration.

The above data indicated that the aptamer could bind with CD19 protein and CD19-expressing lymphoma cells with certain degree of specificity. It is also important to measure the affinity of the aptamer to its target. To quantitatively evaluate the aptamer's binding affinity to CD19, beads coated with CD19 protein were incubated with increasing concentrations of FITC-labeled aptamer, and subsequently analyzed by flow cytometry. Using a non-linear regression analysis, the Kd of the aptamer for binding with CD19 protein was estimated to be 85.4nM (Figure 5).

### Formation of aptamer-doxorubicin complex

**Figure 6:**
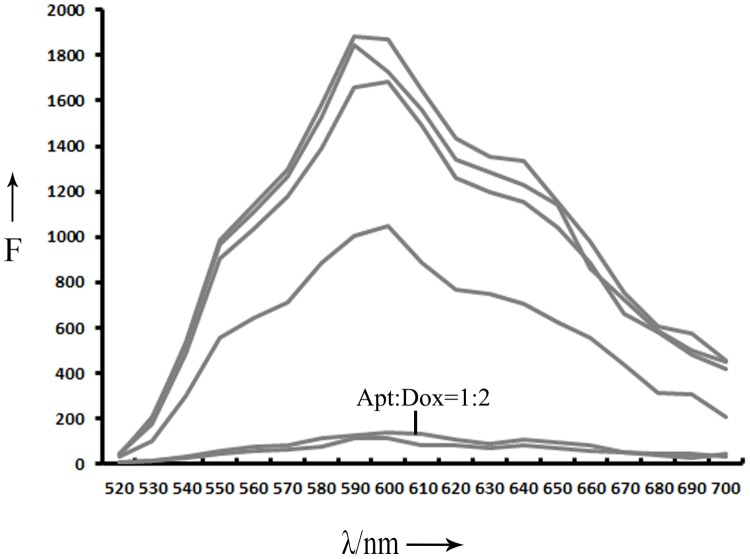
Fluorescence spectra evaluation of Apt-Dox Doxorubicin solution were mixed with increasing molar ratios of the LC1 aptamer (from top to bottom:0, 0.0001, 0.001, 0.005, 0.05, 0.5, and 1).

To explore the possibility of using aptamer LC1 as a targeting ligand in selective drug delivery system, an aptamer-doxorubicin complex (Apt-Dox) was formulated. Previous studies have established that doxorubicin tends to incorporate into DNA structure, and that intercalation of the drug into DNA does not change the capability of aptamers to bind with targets [18, 27]. Doxorubicin normally emits a red fluorescence. However, when it is intercalated into DNA, the fluorescence is quenched. This phenomenon can be utilized to assess the formation of Apt-Dox complex [15, 18, 27]. To calculate the quality of doxorubicin inserted into DNA, various concentrations of the DNA aptamer were mixed with fixed amount of doxorubicin in this study. The fluorescence of the mixture was analyzed by fluorescence spectroscopy (Figure 6). As the aptamer concentration increased, more doxorubicin incorporated into the DNA structure of the aptamers, and the fluorescence of the mixture gradually decreased. The fluorescence was largely quenched when Apt/Dox molar ratio reached 1/2, implying that, at this molar ratio, most drug molecules were inserted into the DNA structure of the LC1 aptamer. The results indicate that each DNA aptamer can carry two doxorubicin molecules on average.

### Apt-Dox was selectively uptaken into CD19-positive lymphoma cells

**Figure 7:**
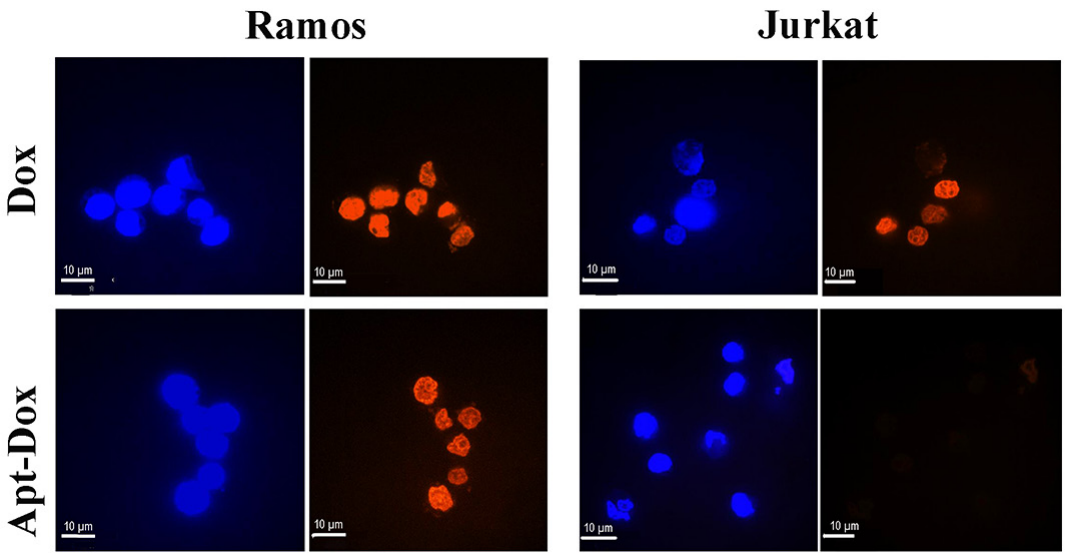
Evaluation of cellular uptake of doxorubicin Confocal microscopy images of CD19-positive Ramos cells and CD19-negative Jurkat cells treated with free Dox (upper panel) or Apt-Dox (lower panel). Free doxorubicin emits a red fluorescence. In the lower panel, the red fluorescence was presumably from free doxorubicin released from Apt-Dox, which was taken up by the cells and digested. The nuclei were stained blue with DAPI.

To investigate whether Apt-Dox could serve as a targeted drug delivery system and accumulate doxorubicin in CD19-positive tumor cells, a drug uptake study was conducted *in vitro*. CD19-positive lymphoma cells (Ramos) and CD19-negative cells (Jurkat) were separately incubated with free Dox or Apt-Dox. Confocal microscopy was used to analyze the fluorescence emitted by doxorubicin. The results were shown in Figure 7. When treated with free Dox, the drug was uptaken by both cell lines (Figure 7, the upper panel), indicating that free Dox unselectively diffused into all cells. However, when treated with Apt-Dox, the drug was mainly taken up by CD19-positive lymphoma cells (Figure 7, the lower panel), suggesting that Apt-Dox could differentiate CD19-positive cells from CD19-negative cells and selectively deliver Dox into the former. The data also implied that the aptamer probably retained its CD19-recognizing capability with Dox inserted in its DNA structure.

**Figure 8:**
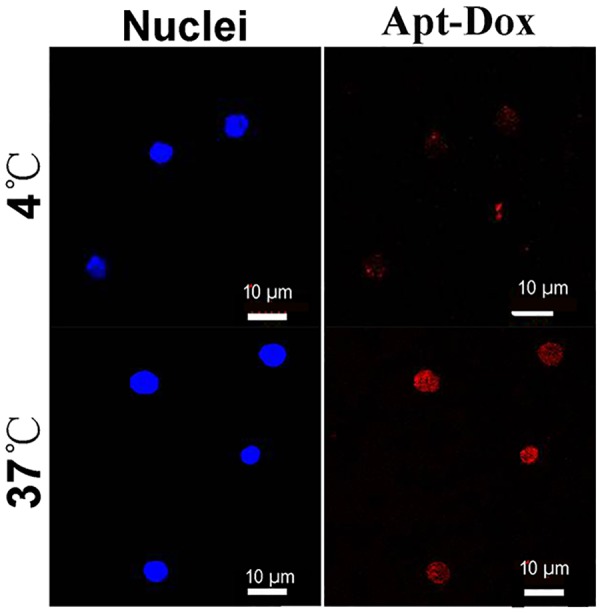
Internalization of Apt-Dox by CD19-positive cells at 37°C and 4°C Ramos lymphoma cells were incubated with Apt-Dox for 2h at 37°C and 4°C, respectively. Confocal microscopy was applied to obtain the cellular images. The red fluorescence was emitted by doxorubicin. The nuclei were stained blue with DAPI.

To evaluate the aptamer internalization by target cells, Apt-Dox was incubated with CD19-positive Ramos cells at 37°C and 4°C, respectively. It is known that cellular endocytosis is largely inhibited at the low temperature of 4°C [14]. The results were presented in Figure 8, while Apt-Dox carried doxorubicin into the Ramos cells at 37°C, it failed to do so at 4C, presumably because the endocytosis of Apt-Dox was largely inhibited at the lower temperature of 4°C.

### Apt-Dox engendered selective cytotoxicity against CD19-positive lymphoma cells

**Figure 9:**
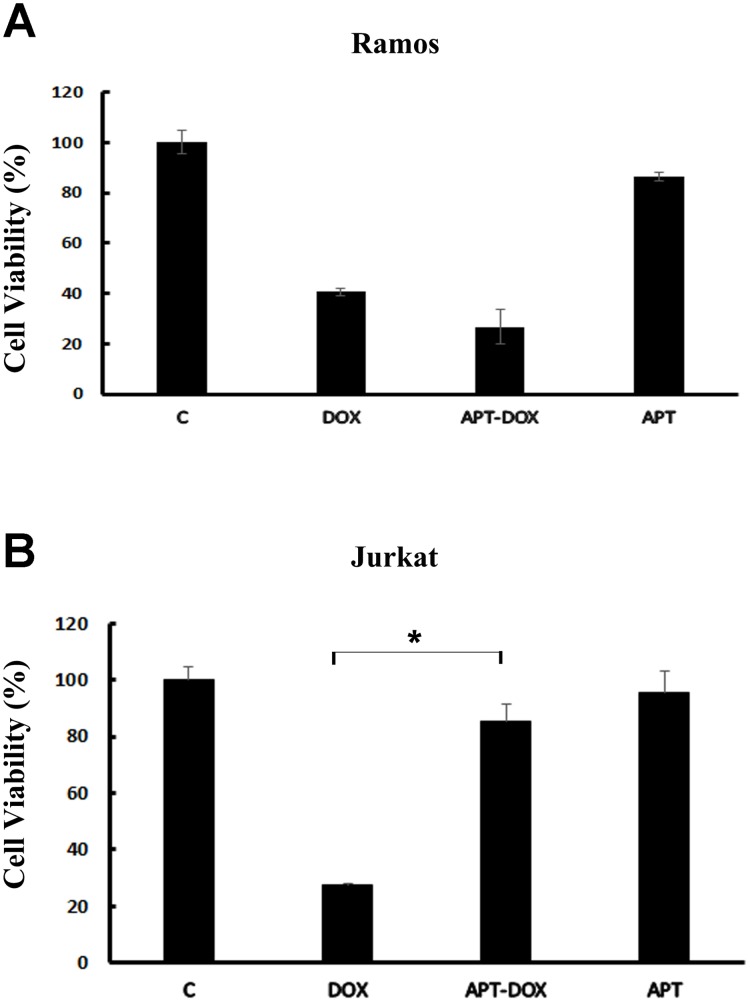
Assays of cytotoxicity generated by Dox or Apt-Dox The CD19-positive Ramos cells and the CD19-negative Jurkat cells were treated with free Dox, Apt-Dox, or free Apt. After 48h of further incubation, the cells were evaluated with standard MTS assay per manufacturer's protocol (mean ± SD, n = 6). The star indicates a statistically significant difference between the Dox and the Apt-Dox groups (p<0.01).

The above results showed that Apt-Dox could selectively carry doxorubicin into CD19-positive cells. However, it was still unknown whether Apt-Dox could generate targeted cytotoxicity to these CD19-positive lymphoma cells. To address this issue, we compared the cytotoxic effects of free Dox, Apt-Dox, and free Apt to CD19-positive (Ramos) and CD19-negative (Jurkat) cells *in vitro*, by implementing a standard MTS assay. As presented in Figure 8, free Dox resulted in similar levels of cytotoxicity to both CD19-positive and -negative cells. Apt-Dox complex, however, mainly produced a robust cytotoxicity towards CD19-positive lymphoma cells (Figure 9A), and significantly reduced the cytotoxicity to CD19-negative cells (Figure 9B, *p*<0.01). Aptamer *per se*, in comparison, caused little cytotoxicity to both cell lines. The results indicated that Apt-Dox generated a CD19-targeted cytotoxicity *in vitro* by selectively suppressing the growth of CD19-positive lymphoma cells.

## DISCUSSION

The main purpose of this study was to develop an aptamer that could bind to CD19, which is an important therapeutic target for treatment of B lymphocytic malignancies. Here in this study, using the SELEX protocol and a CD19 protein as target, we selected a DNA aptamer that could bind with CD19 proteins efficiently. The selected aptamer was 59-base long (Figure 1). It could bind with CD19 with minimal cross reactivity to control proteins such as BSA or OVA (Figure 2). Interestingly, the aptamer could also bind to CD19-positive lymphoma cells, with weak cross-reaction to CD19-negative control cells (Figures 3 & 4). The aptamer bound to the CD19 recombinant protein with a *K*_d_ of 85.4 nM, (Figure 5). An aptamer-drug complex could selectively deliver doxorubicin into CD19-positve lymphoma cells *in vitro*, and reduce the drug intake by CD-19 control cells (Figures 7–9), indicating that the aptamer may potentially serve as a tumor-homing ligand for targeted therapy against lymphocytic malignancies.

CD19 is an attractive target for therapies against lymphocytic malignancies. CD19 has a high homogeneous expression in most cases of lymphoma, and is B-cell lineage–restricted. Recently, CD19-targeted therapy generated unprecedented outcomes in clinical trials. In particular, CD19-targeted car-modified T cell therapy (CAR-T) for refractive ALL achieved remission rates of 70–93% [28], clearly indicating the value of CD19 as an important therapeutic target against lymphocytic malignancies. Currently, CD19-targeted therapy is largely based on antibody technology, in that the targeting ligand for recognizing tumor cells is either an antibody or part of an antibody, such as single-chain variable fragments (scFv) of antibody in case of CAR-T therapy.

In addition to antibodies, aptamers can also function as tumor-targeting ligands. Compared to antibodies, advantages of aptamers include low production cost, easy chemical modification, and low immunogenicity. Aptamers have been shown to bind to cancer cells *in vivo* and selectively deliver therapeutic agents to tumor tissue, with significant improvement of therapeutic efficacy. Giangrande *et al* developed an aptamer-siRNA complex that could enhance the delivery of siRNA to tumor cells and specifically inhibit tumor growth in a xenograft murine model of prostate cancer [29]. Other than applications in targeted tumor therapy, aptamers also have values in construction of targeted imaging contrasts. Zhu et al reported that an aptamer-modified iron oxide nanoparticles could achieve simultaneous contrast enhancement in both T1-and T2-weighted magnetic resonance imaging, and target the cancer stem cells located in hypoxic regions [30]. Moreover, aptamers can also be employed for recognizing tumor markers in histochemistry studies. It has been reported that an DNA aptamer could be effectively applied to evaluate the HER2 expression profile in clinical samples of human breast cancer [31].

Although CD19 is a therapeutic target of known clinical importance, no CD19 aptamer has been reported in literature so far. Here in this study, we developed the first CD19 aptamer that could bind with the extracellular domain of CD19 protein. The aptamer could also distinguish between the CD19-positive and the CD19-negative cells. Moreover, Apt-Dox selectively delivered doxorubicin to CD19-positive lymphoma cells, while reducing the toxicity to CD19-negative control cells. These results indicate that, in addition to therapy based on antibody technology, it is theoretically feasible to develop a new form of CD19-targeted therapy based on aptamer technology.

It is interesting that free doxorubicin diffused into both the CD19-positive lymphoma cells and the CD19-negative control cells (Figure 7 upper panel), while Apt-Dox mainly entered the CD19-positive cells (Figure 7 lower panel). We hypothesized that free doxorubicin, due to its lipophilic nature, readily diffused into both types of cells, and therefore did not have a targeting preference. In contrast, Apt-Dox was formed by inserting doxorubicin into the DNA structure of the aptamer. This aptamer-drug complex could not freely diffuse into cells, due to DNA's negative charge and hydrophilic nature. The reason Apt-Dox could enter CD19-positive lymphoma cells was presumably that the aptamer recognized and bound to the CD19 structure on CD19-positive cells, resulting in the endocytosis of the Apt-Dox complex. Apparently, much future research is warranted to delineate the detailed mechanism by which Apt-Dox entered CD19-positive lymphoma cells.

Future research may also attempt to conjugate the thioaptamer to imaging contrasts or anticancer agents, to construct aptamer-guided diagnostic or therapeutic systems. DNA aptamers made of regular oligonucleotides are susceptible to nuclease digestion and may lose their binding functionality. Chemical modification of aptamers may potentially solve this problem. Specifically, the phosphate backbone of DNA can be modified with phosphorothioate to generate thioaptamers, which have enhanced nuclease resistance. Such a thioaptamer may be conjugated to a drug-carrying nanoparticle, in order to develop a selective drug delivery system for targeted cancer therapy.

In summary, this study shows that a DNA aptamer can be selected against recombinant CD19 protein. The aptamer has the capability to preferentially bind with CD19-expressing lymphoma cells (Ramos and Raji) *in vitro*. Such an aptamer may have theoretical utility as a tumor-homing ligand in targeted therapy against CD19-expressing cancers.

## MATERIALS AND METHODS

Oligonucleotide primers were synthesized by Invitrogen (Shanghai China). Recombinant human CD19 protein and anti-CD19 antibody were purchased by Abcam (UK). Bovine serum albumin (BSA) was purchased from Tbdscience (Tianjin China). Mono-dispersed magnetic urea-formaldehyde microspheres were purchased from Baseline Chromtech (Tianjin China). Ovalbumin (OVA) was purchased from Amresco (US). Streptavidin-coated magnetic beads were purchased from Promega (US).

### Tumor cell lines

The Cell lines, Raji (human B-cell lymphoma), Ramos (human B-cell lymphoma), Jurkat (human T-cell lymphoma), and NB-4 (Acute promyelocytic leukemia) were purchased from the Cell Resource Center of the Chinese Academy of Medical Sciences (Beijing, China). Raji, Ramos, Jurkat, and NB-4 cells were maintained in RPMI-1640 medium supplemented with 100 U/mL penicillin, 100 μg/mL streptomycin, and 10% fetal bovine serum (FBS, Gibico). All cells were grown at 37°C, 5% CO2.

### The selection DNA library and primers

A random DNA library pool consisted of 59 nucleotides, containing a 21-nt central random region flanked by two different sequences of 19 nt for PCR amplification. The library sequence was 5’- TGCGTGTGTAGTGTGTCTG-N21-CTCTTAGGGATTTGGGCGG-3’. A FITC- labeled 5’ primer P1 (5’-FITC- TGCGTGTGTAGTGTGTCTG -3’) was used to monitor the enrichment of aptamer during the selection process. A biotinylated 3’ primer P2 (5’-biotin- CCGCCCAAATCCCTAAGAG -3’) was used in PCR to generate double strand DNA (dsDNA) and for separation of ssDNA from dsDNA. Unlabeled P1 and P2 primers were used for cloning of the enriched sequences.

### The target protein

The target protein for aptamer selection is recombinant human CD19 protein. The CD19 protein was immobilized to magnetic beads by the reaction of –Epoxy and –NH2. CD19 protein (2 μg) was mixed with magnetic beads and reacted for 24 h in carbonate buffer solution(PH=10.7) at room temperature with gentle shaking. The CD19-coated magnetic beads were then washed for three times and resuspended in PBS. Similar method was applied to conjugate the beads with other molecular, such as BCA, OVA.

### The selection process of aptamers

The procedures of selection were as follows. To reduce the nonspecific binding with blank beads, 50 pmol of random ssDNA pool was incubated with blank beads at 37°C for 0.5 h with shaking. The magnetic beads were pulled down by a magnetic field. The unbound ssDNA was collected from the supernatant, and incubated with beads coated with CD19 protein at 37°C for 30 min with gentle shaking for positive selection. After incubation, CD19-coated beads were washed with 500 μl of binding buffer for three times. Subsequently, bead-bound oligonucleotides mixtures were PCR amplified (30 cycles of 40 sec at 94°C, 30 sec at 65°C, 2 min at 72°C, followed by 10 min at 72°C) in a reaction buffer containing dATP, dCTP, dGTP and dTTP (200μM), MgCl_2_(2 mM), FITC- or biotin-labeled primers and the Taq polymerase (0.5 U). After the application, biotinylabeled dsDNA was mixed with streptavidin coated magnetic beads for 15 min at room temperature, and then washed three times with PBS. The FITC-labeled single-strand aptamers were separated from the biotin- and FITC-labeled dsDNA using a 5 min incubation of 50 μl of 0.1 M NaOH. The FITC-labeled aptamers were removed within a magnetic field and used for the next round of SELEX. After 8 rounds of selection, the selected ssDNA pool was PCR-amplified using unmodified primers and cloned into Escherichia Coli with the TA cloning kit for DNA sequencing. The secondary structure of the aptamer was predicted by the mfold web server [32].

### Flow cytometry analysis

To evaluate the enrichment of aptamer candidates after each round of selection, the FITC-labeled ssDNA pool was incubated with CD19-coated magnetic beads in PBS buffer with gentle shaking for 30 min. The beads were then washed three times with PBS and re-suspended in PBS. The fluorescence signal was analyzed with a FACScalibur cytometer (Accuri C6, US). The FITC-labeled randomized ssDNA library was used to generate the control signal.

The binding affinity of aptamers was quantified by flow cytometry. Varying concentrations of FITC-labeled aptamers were incubated with the CD19-coated magnetic beads in PBS at 37°C for 30 min. The beads were washed three times with PBS, and then suspended in PBS. The fluorescence signal was analyzed by flow cytometry. The FITC-labeled random ssDNA pool was used as a background control to determine nonspecific binding. All of the experiments for binding assay were repeated three times. The mean fluorescence intensity of aptamers bound to target-coated beads was used to calculate the Kd by subtracting the mean fluorescence intensity generated by nonspecific binding. The equilibrium dissociation constants (*K*_d_) was obtained by fitting the dependence of fluorescence intensity of specific binding on the concentration of the aptamers to the equation Y= B max X/ (Kd + X), where Y represented the reciprocal of the average fluorescence intensity, X represented the reciprocal of aptamer's concentration, and Bmax represented the maximum binding capacity of aptamer bound to CD19.

The cellular binding experiment was performed by flow cytometric analysis (FCM) and confocal microscopy (Perkin Elmer Ultraview, US). For FCM analysis, the FITC-labeled aptamer was incubated with 10^^5^ of either Raji, Ramos, Jurkat, or NB-4 cells in PBS at 37°C for 30 min with gentle shaking. Cells were then washed three times with PBS and analyzed with flow cytometry.

#### Confocal Imaging studies

Cy3-labeled aptamer was incubated with 10^^5^ of either Raji, Ramos, Jurkat, or NB-4 cells in PBS at 37°C for 30 min with gentle shaking. After washing with PBS for three times, the cells were fixed by 4% formaldehyde for 10min, and incubated with anti-human CD19 antibodies in PBS at 4°C for 1h. After washing with PBS for three times, the cells were incubated with a secondary antibody labeled with Alexa Fluor 488 in PBS at 4°C for 1h. After washing with PBS for three times, the cells were mounted onto a glass slide. Ten microliters of DAPI was added to the cells, and a coverslip were seated on top of the cells. Confocal microscopy scanning microscopy was applied to evaluate the fluorescence images.

### Loading aptamers with doxorubicin

Aptamers were heated at 95°C for 5min and cooled on the ice for 15min immediately. A fixed concentration of doxorubicin (3nM) was mixed with varying concentrations of the aptamer LC1 at 37°C for 1h. The molar ratios of aptamer/Dox were 0, 0.0001, 0.005, 0.01, 0.5, and 1 respectively. The fluorescence spectrum of doxorubicin was examined in black 96-well plate by a Synergy4 analyzer (λEx = 488 nm, λEM = 520-700 nm).

### Cellular uptake studies

The cellular uptake of Apt-Dox or free doxorubicin was studied by confocal fluorescence scanning microscopy (Perkin Elmer Ultraview, US). Ramos cells or Jurkat cells (2^*^10^^5^) were incubated with 3μM free Dox or Apt-Dox complex for 3h at 37°C and washed twice with PBS. The cells were then fixed with 4% formaldehyde for 10 min at 4°C, washed twice with PBS. Ten microliters of DAPI was added to the cells and mixed them. The mixture was added to the slide, and then a glass coverslip with cells was sealed and stained for 5 minutes. Confocal fluorescence scanning microscopy was used to analyzed the cell fluorescence.

To evaluate the aptamer internalization by target cells, 3μM Apt-Dox was incubated with CD19-positive Ramos cells at 37°C or 4°C for 2 hours and washed three times with PBS. The cells were then fixed with 4% formaldehyde for 10 min at 4°C and washed twice with PBS. Ten microliters of DAPI was added to the cell suspension, which was loaded onto a glass slide. The cells were covered with a glass coverslip and stained for 5 minutes. Confocal fluorescence scanning microscopy was applied to obtain the fluorescence image of the cells.

### *In vitro* cytotoxicity

To evaluate the cytotoxic effects of Apt-Dox or Dox against Ramos and Jurkat cells, both cell lines were grown in 96-well plates. The cells were treated with 3μM Dox, Apt-Dox, or aptamer only at 37°C for 4 hours. The cells in plate were centrifuged and washed twice with PBS, and cultured for an additional 48 hours. MTS assay (Promega, US) was used to determine the cell viability according to the standard protocol as outlined by the manufacture.

### Statistics

Statistical analysis was performed with the statistical SPSS 13.0 software. The nonparametric test was used to calculate the probability of significant differences among the groups. Statistical significance was defined as p<0.05.
